# Estimation of a Human-Maneuvered Target Incorporating Human Intention

**DOI:** 10.3390/s21165316

**Published:** 2021-08-06

**Authors:** Yongming Qin, Makoto Kumon, Tomonari Furukawa

**Affiliations:** 1 Department of Electrical and Computer Engineering, University of Virginia, Charlottesville, VA 22903, USA; yq9vc@virginia.edu; 2 Faculty of Advanced Science and Technology, International Research Organization of Advanced Science and Technology, Kumamoto University, 2-39-1 Kurokami, Chuo-ku, Kumamoto 860-8555, Japan; kumon@gpo.kumamoto-u.ac.jp; 3 Department of Mechanical Engineering, University of Virginia, Charlottesville, VA 22903, USA

**Keywords:** estimation, tracking, human intention, motion pattern, prediction, multiple model, interacting multiple model, multirotor, drone

## Abstract

This paper presents a new approach for estimating the motion state of a target that is maneuvered by an unknown human from observations. To improve the estimation accuracy, the proposed approach associates the recurring motion behaviors with human intentions, and models the association as an intention-pattern model. The human intentions relate to labels of continuous states; the motion patterns characterize the change of continuous states. In the preprocessing, an Interacting Multiple Model (IMM) estimation technique is used to infer the intentions and extract motions, which eventually construct the intention-pattern model. Once the intention-pattern model has been constructed, the proposed approach incorporate the intention-pattern model to estimation using any state estimator including Kalman filter. The proposed approach not only estimates the mean using the human intention more accurately but also updates the covariance using the human intention more precisely. The performance of the proposed approach was investigated through the estimation of a human-maneuvered multirotor. The result of the application has first indicated the effectiveness of the proposed approach for constructing the intention-pattern model. The ability of the proposed approach in state estimation over the conventional technique without intention incorporation has then been demonstrated.

## 1. Introduction

Most dynamic targets to track or engage are either human-maneuvered or humans themselves. Estimating the state of such a human-maneuvered target is essential and important, and has attracted tremendous interest in the last decades [1,2,3,4]. Despite the importance, difficulty in the estimation of the human-maneuvered target lies in the motion uncertainty. Even though the motion model of the target may be well or precisely known, the control of the human is often unknown [5]. The motion, as a result, becomes considerably different from the expectation. This gives rise to need for the ability to handle motion uncertainty [6].

For a human-maneuvered target, estimation techniques proposed in the past to handle motion uncertainty can be classified into two types. In the first, a single accurate motion model is developed and used to describe the motion behavior. Due to their robust estimation upon past observations, various Bayesian methods, including the parametric Kalman filters (KFs) and the nonparametric particle filters, have been applied by characterizing the estimation problem and identifying the best estimation technique for the problem [7,8,9,10,11,12,13]. Steckenrider and Furukawa [14] proposed to introduce higher-order terms to the motion model through Taylor series expansion and adaptively estimated the target state. Gindele et al. [15] improved the motion model by incorporating the situational context and extending the state space. As human control is unknown most of the time, conservative motion behaviors such as constant velocity (CV) and constant acceleration (CA) have been incorporated as the most probable human controls [5]. Instead of the motion model, Mehra [16] estimated the covariances of motion noise and observation noise when the filter is detected not working optimally. Almagbile et al. [17] evaluated three adaptation methods of noise covariances and showed improvements over the conventional Kalman filter. It is effective to control uncertainty when the deterministic motion accuracy can no longer be improved. In addition to the model and its uncertainty, other work has dealt with unknown human control and its uncertainty from the motion noise [5]. The human control dominates the motion behavior when the target has a large unconstrained workspace. Bogler et al. [18] represented the time-varying human control deterministically by piecewise constants and estimated the control in addition to the state. Chakrabarty et al. [19] assumed the exogenous input and its derivative to be bounded for a class of nonlinear systems in state estimation. Conte and Furukawa [20] used head motion as an additional indicator when the target is a human and improved the estimation accuracy. While they are more detailed and more adaptively represented, these motion models cannot keep capturing the target motion and estimating its state well particularly if the motion is drastically changed by a human. This is due to the limited representation of a single model.

In the second, multiple models, which are either superposed or switched, have been used to estimate more varying motion behavior [21,22,23,24,25]. The multiple-model (MM) estimation methods extend existing techniques to handle multiple models and cover a wider range of motion behavior [26]. Henk et al. [21] proposed the interacting MM (IMM) method that uses a fixed set of motion models with Markovian switching coefficients. The transition probability and model likelihood were introduced to recursively adapt the model probabilities. Li et al. [27] proposed the variable-structure MM (VSMM) method to overcome the limitations of using a fixed set of models in describing the motion. The VSMM method introduces model set adaption besides the model adaptation and thus can describe and estimate even a broader range of motion behavior. Recently, Xu et al. [28] has engaged with estimating varying motion behaviors by adapting parameters where a fixed coarse grid and an adaptive fine grid of the parameters were combined to determine the models that best match the target motion behavior. Despite the wider covering, it is still insufficient to capture and estimate the target if the human control changes considerably. The MM methods are rather formulated to cover a larger state space given the most probable human control. As the drastic control change may magnify changes in state space, the resulting target state could be beyond the permissible space of the MM estimation. In addition, the use of the deterministic control makes the estimation underestimated as the human control is most uncertain.

This paper presents an approach for estimating the state of a human-maneuvered target by associating the recurring motion behaviors with human intentions. The proposed approach consists of a preprocess, which constructs the so-called intention-pattern model to encapsulate the human intention, and the main process, which allows state estimation using the intention-pattern model. In the preprocess, the intention-pattern model is constructed from the prior observations by running a revised IMM estimation, extracting motion behaviors of each human intention, aligning them, and probabilistically representing its behavior. The main process, then, uses standard state estimation such as KF extensively using the probabilistically represented intention-pattern model. The strength of the proposed approach lies in the incorporation of the intention-pattern model as the incorporation can make the estimation not only accurate in mean but also precise in covariance.

The paper is organized as follows. The next section describes the estimation problem and its solution using the IMM estimation method, which is not only a generalized formulation but also the technique used in the preprocess of the proposed approach. Section 3 presents the proposed estimation approach including the preprocess and the main process. Numerical validation investigating the effectiveness of both the intention-pattern model and the state estimation is presented in Section 4. Conclusions are summarized in the final section.

## 2. Estimation of a Human-Maneuvered Target Using IMM Estimation

### 2.1. Estimation Problem Formulation

**Figure 1:**
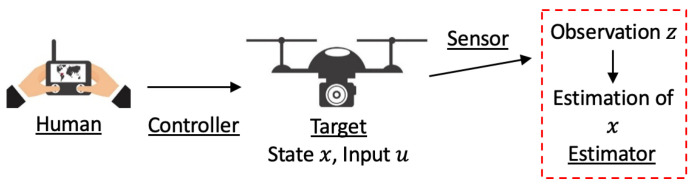
The problem of estimating a human-maneuvered target from observations.

Figure 1 shows a schematic diagram of the problem of estimating the state of a human-maneuvered target in case the target is a multirotor. When maneuvering a target, a human operator interacts with a controller using an interface device such as a vehicle panel or a joystick. The controller may be implemented in the interface device, in the target, or both. Some parameters of the controller, such as the maximum speed of the target, are usually configurable to realize different motion behavior. The information of human operation and configurable parameters are not known as no communication with the target is available. The estimator does not affect the human operator and target as well. Having the target observed in the field of view (FOV) of a fixed sensor such as a stereo camera, the goal of the problem is to design the estimator to estimate the target state from observations. The discrete motion model of the target and the observation model are generically given by

(1) $$\begin{array}{cc} x_{k} & =f(x_{k-1},u_{k},w_{k}) \end{array}$$

(2) $$\begin{array}{cc} z_{k} & =h(x_{k},v_{k}) \end{array}$$

where f and h are the motion and the observation models, respectively; $x_{k}$ is the state of the target at step *k* to estimate; $u_{k}$ is the input; $z_{k}$ is the observation; and $w_{k}$ and $v_{k}$ are the motion and observation noises, respectively. Because it is a target maneuvered by a human, f and $w_{k}$ may not be well known, while $u_{k}$ is fully unknown. On the other hand, h and $v_{k}$ are fully known as they are with the sensor(s) of the estimator. With short time interval, it is valid to assume that $w_{k}$ and $v_{k}$ are Gaussian. The problem is resultantly defined as the estimation of $x_{k}$ with no knowledge on $u_{k}$ and some knowledge on f and $w_{k}$, given h, $z_{k}$ and $v_{k}$.

### 2.2. IMM Estimation

Lacking information of f and $u_{k}$ results in high motion uncertainty. The MM estimation methods deal with motion uncertainty by describing the motion with several motion behaviors called modes, which are denoted by $S=\{s^{j}\},j\in N$ where N represents all natural integers. With the definition, a mode $s^{j}$ is used to represent the motion at step *k* when it approximates the motion behavior well, i.e., $s_{k}=s^{j}$. To describe the behavior with minimum complexity, a mode $s^{j}$ is most commonly described by a mathematical model $m^{i}$, which is collectively represented by $M=\{m^{i}\},i\in N$. A model $m^{i}$ thus represents the motion behavior at a step, i.e., $s_{k}=s^{j}=m^{i}$. Li et al. [29] is referred for more description. Most MM estimation methods utilize models of known motion behaviors with different parameters such as variants of the CV and CA models. As an example, suppose that f of Equation (1) at step *k* is approximated by a single linear Gaussian model. The motion model $m^{i}$ is given by

(3) $$\begin{array}{cc} x_{k} & =A_{k}^{\left(i\right)}x_{k-1}+B_{k}^{\left(i\right)}u_{k}^{\left(i\right)}+w_{k}^{\left(i\right)}. \end{array}$$

where $A_{k}^{\left(i\right)}$ is a system matrix, $B_{k}^{\left(i\right)}$ is a control matrix, and $w_{k}^{\left(i\right)}$ is Gaussian with mean 0 and covariance $Q_{k}^{\left(i\right)}$. The symbol ${}^{\left(i\right)}$ indicates that the model $m^{i}$ is used. The observation model (2) is also supposed to be linear Gaussian:

(4) $$\begin{array}{c} z_{k}=C_{k}x_{k}+v_{k} \end{array}$$

where $C_{k}$ is the observation matrix, and $v_{k}$ is Gaussian with mean 0 and covariance $R_{k}$.

**Figure 2:**
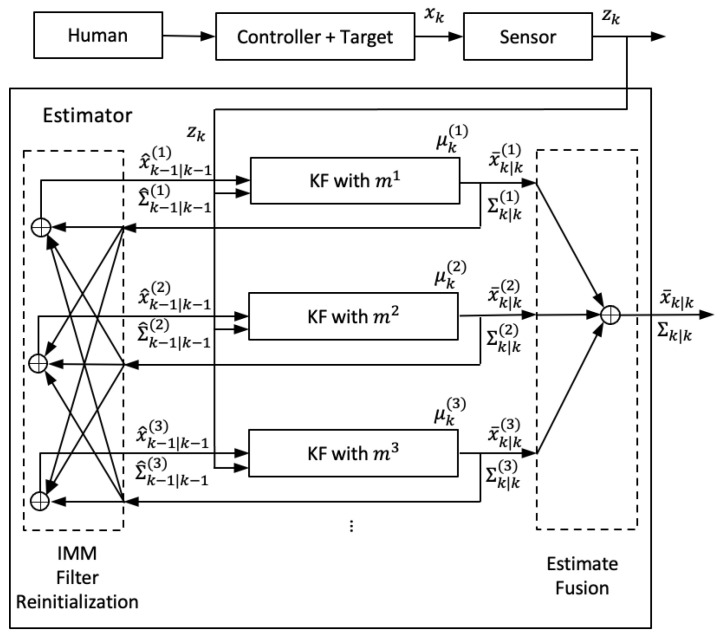
The IMM estimation method.

Figure 2 shows the framework of the IMM estimation method to estimate the target state. The motion behavior is described with a set of models $\left\{m^{1},m^{2},m^{3},...\right\}$. Having the observation $z_{k}$ of the state $x_{k}$, KF is applied for each model (3) with the linear Gaussian assumption. Each KF updates the target state of mean ${\hat{\bar{x}}}_{k-1|k-1}^{\left(i\right)}$ and covariance ${\hat{\Sigma }}_{k-1|k-1}^{\left(i\right)}$ to mean ${\bar{x}}_{k|k}^{\left(i\right)}$ and covariance $\Sigma_{k|k}^{\left(i\right)}$. Each ${\hat{\bar{x}}}_{k-1|k-1}^{\left(i\right)}$ and ${\hat{\Sigma }}_{k-1|k-1}^{\left(i\right)}$ are derived from the IMM filter re-initialization incorporating all of ${\bar{x}}_{k-1|k-1}^{\left(i\right)}$ and $\Sigma_{k-1|k-1}^{\left(i\right)}$. The output ${\bar{x}}_{k|k}$ and covariance $\Sigma_{k|k}$ are calculated by the estimate fusion of all of ${\bar{x}}_{k|k}^{\left(i\right)}$ and $\Sigma_{k|k}^{\left(i\right)}$.

The mathematical derivation is as follows. The event that the model $m^{i}$ matches the mode at step *k* is denoted as $m_{k}^{\left(i\right)}≜\left\{s_{k}=m^{i}\right\}$. The probability of $m_{k}^{\left(i\right)}$ is denoted as $\mu_{k}^{\left(i\right)}≜Pr\left\{m_{k}^{\left(i\right)}\right\}$ where Pr{·} indicates the probability of an event. The IMM estimator assumes the probability of transitioning from a model $m^{i}$ at step *k* to a model $m^{j}$ at step k+1 is constant and known as $\pi_{ij}$:

(5) $$\begin{array}{c} Pr\left\{m_{k+1}^{\left(j\right)}|m_{k}^{\left(i\right)}\right\}=Pr\left\{s_{k+1}=m^{j}|s_{k}=m^{i}\right\}=\pi_{ij}, \end{array}$$

where i,j∈N. For one cycle, the predicted model probability, ${\hat{\mu }}_{k|k-1}^{\left(i\right)}$, is given by

(6) $$\begin{array}{cc} {\hat{\mu }}_{k|k-1}^{\left(i\right)} & ≜Pr\left\{m_{k}^{\left(i\right)}|z_{1:k-1}\right\}=\sum_{j}\mu_{k-1}^{\left(j\right)}\pi_{ji} \end{array}$$

where $z_{1:k-1}$ are the observations from step 1 to step k−1. The weight that $m_{k-1}^{\left(j\right)}$ contributes to $m_{k}^{\left(i\right)}$ is derived as

(7) $$\begin{array}{c} \mu_{k-1}^{j|i}≜Pr\left\{m_{k-1}^{\left(j\right)}|m_{k}^{\left(i\right)},z_{1:k-1}\right\}=\mu_{k-1}^{\left(j\right)}\pi_{ji}/{\hat{\mu }}_{k|k-1}^{\left(i\right)} \end{array}$$

The KF of each model starts with the derivation of input:

(8a) $$\begin{array}{c} {\hat{\bar{x}}}_{k-1|k-1}^{\left(i\right)}≜E\left[x_{k-1}|m_{k}^{\left(i\right)},z_{1:k-1}\right]=\sum_{j}{\bar{x}}_{k-1|k-1}^{\left(j\right)}\mu_{k-1}^{j|i}, \end{array}$$

(8b) $$\begin{array}{c} {\hat{\Sigma }}_{k-1|k-1}^{\left(i\right)}=\sum_{j}\left[\Sigma_{k-1|k-1}^{\left(j\right)}+\left({\bar{x}}_{k-1|k-1}^{\left(i\right)}-{\hat{x}}_{k-1|k-1}^{\left(j\right)}\right){\left({\bar{x}}_{k-1|k-1}^{\left(i\right)}-{\hat{x}}_{k-1|k-1}^{\left(j\right)}\right)}^{⊤}\right]\mu_{k-1}^{\left(j|i\right)}. \end{array}$$

According to the KF formulation, the predicted mean and covariance are derived as

(9a) $$\begin{array}{c} {\bar{x}}_{k|k-1}^{\left(i\right)}=A_{k}^{\left(i\right)}{\hat{x}}_{k-1|k-1}^{\left(i\right)}+B_{k}^{\left(i\right)}u_{k}^{\left(i\right)}, \end{array}$$

(9b) $$\begin{array}{c} \Sigma_{k|k-1}^{\left(i\right)}=A_{k}^{\left(i\right)}{\hat{\Sigma }}_{k-1|k-1}^{\left(i\right)}{\left(A_{k}^{\left(i\right)}\right)}^{⊤}+Q_{k}^{\left(i\right)}. \end{array}$$

For correction, the KF gain is first computed through

(10) $$\begin{array}{c} K_{k}^{\left(i\right)}=\Sigma_{k|k-1}^{\left(i\right)}{\left(C_{k}\right)}^{⊤}{\left(S_{k}^{\left(i\right)}\right)}^{-1} \end{array}$$

where the residual covariance is given by

(11) $$\begin{array}{c} S_{k}^{\left(i\right)}=C_{k}\Sigma_{k|k-1}^{\left(i\right)}{\left(C_{k}\right)}^{⊤}+R_{k} \end{array}$$

The corrected mean and covariance are derived as

(12a) $$\begin{array}{c} {\bar{x}}_{k|k}^{\left(i\right)}={\bar{x}}_{k|k-1}^{\left(i\right)}+K_{k}^{\left(i\right)}{\tilde{z}}_{k}^{\left(i\right)}, \end{array}$$

(12b) $$\begin{array}{c} \Sigma_{k|k}^{\left(i\right)}=\left(I-K_{k}^{\left(i\right)}C_{k}\right)\Sigma_{k|k-1}^{\left(i\right)}, \end{array}$$

where the observation residual is given by

(13) $$\begin{array}{c} {\tilde{z}}_{k}^{\left(i\right)}=z_{k}-C_{k}{\bar{x}}_{k|k-1}^{\left(i\right)} \end{array}$$

For IMM estimation, the model likelihood $L_{k}^{\left(i\right)}$ is assumed as

(14) $$\begin{array}{c} L_{k}^{\left(i\right)}≜Pr\left\{{\tilde{z}}_{k}^{\left(i\right)}|m_{k}^{\left(i\right)},z_{1:k-1}\right\}\\ \overset{\mathrm{assume}}{=}{\left|2\pi S_{k}^{\left(i\right)}\right|}^{-\frac{1}{2}}\exp\left[-\frac{1}{2}{\left({\tilde{z}}_{k}^{\left(i\right)}\right)}^{⊤}{\left(S_{k}^{\left(i\right)}\right)}^{-1}{\tilde{z}}_{k}^{\left(i\right)}\right] \end{array}$$

and the model probability $\mu_{k}^{\left(i\right)}$ is given by

(15) $$\begin{array}{c} \mu_{k}^{\left(i\right)}≜Pr\left\{m_{k}^{\left(i\right)}|z_{1:k}\right\}=\frac{{\hat{\mu }}_{k|k-1}^{\left(i\right)}L_{k}^{\left(i\right)}}{\sum_{j}{\hat{\mu }}_{k|k-1}^{\left(j\right)}L_{k}^{\left(j\right)}}. \end{array}$$

The overall mean and covariance are derived as

(16a) $$\begin{array}{c} {\bar{x}}_{k|k}≜E\left[x_{k}|z_{1:k}\right]=\sum_{i}{\bar{x}}_{k|k}^{\left(i\right)}\mu_{k}^{\left(i\right)} \end{array}$$

(16b) $$\begin{array}{c} \Sigma_{k|k}=\sum_{i}\left[\Sigma_{k|k}^{\left(i\right)}+\left(x_{k|k}^{\left(i\right)}-{\bar{x}}_{k|k}\right){\left(x_{k|k}^{\left(i\right)}-{\bar{x}}_{k|k}\right)}^{⊤}\right]\mu_{k}^{\left(i\right)}. \end{array}$$

Owing to the introducing of transition probability $Pr\{m_{k+1}^{\left(j\right)}|m_{k}^{\left(i\right)}\}$ of Equation (5) and the likelihood $L_{k}^{\left(i\right)}$ of Equation (14), the model probabilities $\mu_{k}^{\left(i\right)}$ adapt to match the current motion. Suppose the model $m^{\left(i\right)}$ matches the current mode better, the filter of $m^{\left(i\right)}$ contributes more on ${\bar{x}}_{k|k}$ and $\Sigma_{k|k}$ by having a higher model probability $\mu_{k}^{\left(i\right)}$.

For estimation with one motion model, the single motion model (1) cannot impair its inconsistency from the actual motion when the human has changed the target motion considerably. The IMM method estimates in a larger state space due to the usage of multiple models, but it still uses the most probable deterministic human control such as the CV and CA. If the control is different, the multiple models of the IMM method may not be able to cover the space of estimation and could lead to a wrong estimation. The uncertainty could also be underestimated since the unknown human control, which is most uncertain, is handled deterministically. This limitation of the conventional techniques affects the quality of estimation when the target is human-maneuvered.

## 3. Proposed Approach Using Intention-Pattern Model

As the contributions of this paper are the construction of a intention-pattern model and the state estimation using the intention-pattern model, this section describes each contribution in a subsection. Section 3.1 presents the overview of the construction of an intention-pattern model, followed by the details of the two major components, which are the intention inference and the intention-pattern modeling. The implementation of the constructed intention-pattern models into the state estimation is then detailed in Section 3.2.

### 3.1. Construction of Intention-Pattern Model

#### 3.1.1. Overview

**Figure 3:**
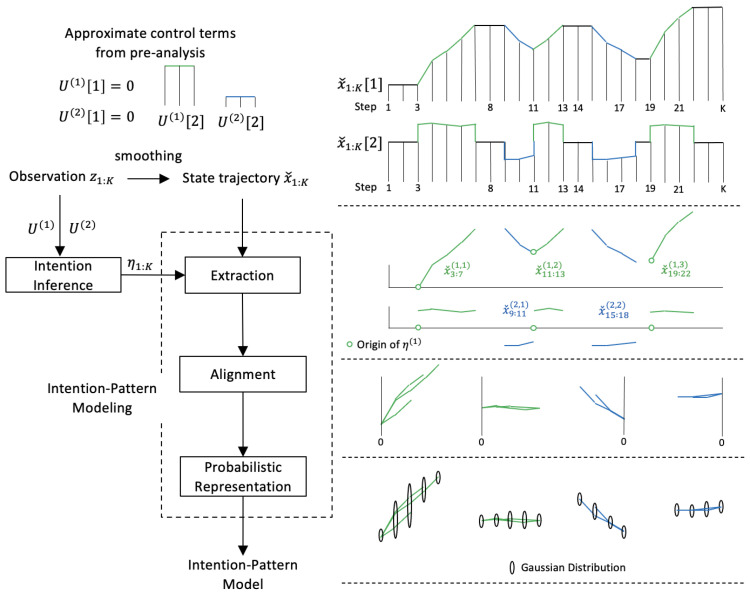
Construction of intention-pattern model. (·)[i] represents the *i*th dimension of (·).

Figure 3 shows the construction of the intention-pattern model where an example illustration is given on the right side. The prior analysis of the target behavior leads to the extraction of a set of human intentions $H=\{\eta^{\left(i\right)}|\forall i\}$ and the corresponding approximate control terms, $U^{\left(i\right)}$. Each human intention $\eta^{\left(i\right)}$ is an expression describing an aim or a plan, such as “moving forward” and “turning right”.

Each human intention at step *k* is defined as a function of the recent states of $N_{h}$ steps:

(17) $$\begin{array}{c} \eta_{k}=\alpha \left(x_{k-N_{h}+1:k}\right). \end{array}$$

Compared with the human actions which are of one step and are associated with the control input $u_{k}$, the human intentions are labels of continuous states of multiple steps.

The corresponding control term could vary, but let it be constant for simplicity. Because the intention is defined for a period, the figure illustratively shows each control term with two steps. Given a sequence of observations $z_{1:K}$, the human intention at step *k*, $\eta_{k}=\eta^{\left(i\right)}\in H$, is first inferred for all steps, i.e., $\eta_{1:K}=\left\{\eta_{1},...,\eta_{K}\right\}$. We assume that the observations are fully observable for simplicity. After smoothing the observations and deriving the state trajectory ${\overset{ˇ}{x}}_{1:K}$, the proposed construction technique identifies segments in state space exhibiting the extracted intention, ${\overset{ˇ}{x}}_{k_{s(i,j)}:k_{e(i,j)}},j\in N$, where $k_{s(i,j)}$ and $k_{e(i,j)}$ are the starting and the ending steps. Three segments of intention $\eta^{\left(1\right)}$ and two segments of intention $\eta^{\left(2\right)}$ are shown in the example illustration. The segments of the same intention are aligned to characterize the pattern of motion probabilistically. The intention-pattern model, describing the relationship between the input intention and the output motion pattern, is finally represented by a set of Gaussian distributions.

#### 3.1.2. Intention Inference

As states $x_{k-N_{h}+1:k}$ are not directly available, this section proposes the approach to infer the human intention $\eta_{k}$ based on observations. Given observations $z_{1:k}$ in addition to the control terms corresponding to the extracted intentions, $U^{\left(i\right)},\forall i$, the first process of the intention inference is to run the IMM estimation. There is only one motion model, but the motion is simulated for each intention $U^{\left(i\right)}$:

(18) $$x_{k}=A_{k}x_{k-1}+U^{\left(i\right)}+w_{k},$$

where $A_{k}$ and $w_{k}$ are determined from the analysis of target motion. Having Equation (4) as an observation model, the KF updates the mean $x_{k|k}$ and the covariance $\Sigma_{k|k}$ similarly to Equations (9)–(13) for each intention:

(19a) $$\begin{array}{c} {\bar{x}}_{k|k-1}^{\left(i\right)}=A_{k}{\bar{x}}_{k-1|k-1}^{\left(i\right)}+U^{\left(i\right)} \end{array}$$

(19b) $$\begin{array}{c} \Sigma_{k|k-1}^{\left(i\right)}=A_{k}\Sigma_{k-1|k-1}^{\left(i\right)}{\left(A_{k}\right)}^{⊤}+Q_{k}. \end{array}$$

(19c) $$\begin{array}{c} K_{k}^{\left(i\right)}=\Sigma_{k|k-1}^{\left(i\right)}{\left(C_{k}\right)}^{⊤}{\left(S_{k}^{\left(i\right)}\right)}^{-1} \end{array}$$

(19d) $$\begin{array}{c} S_{k}^{\left(i\right)}=C_{k}\Sigma_{k|k-1}^{\left(i\right)}{\left(C_{k}\right)}^{⊤}+R_{k} \end{array}$$

(19e) $$\begin{array}{c} {\tilde{z}}_{k}^{\left(i\right)}=z_{k}-C_{k}{\bar{x}}_{k|k-1}^{\left(i\right)}, \end{array}$$

(19f) $$\begin{array}{c} {\bar{x}}_{k|k}^{\left(i\right)}={\bar{x}}_{k|k-1}^{\left(i\right)}+K_{k}^{\left(i\right)}{\tilde{z}}_{k}^{\left(i\right)}, \end{array}$$

(19g) $$\begin{array}{c} \Sigma_{k|k}^{\left(i\right)}=\left(I-K_{k}^{\left(i\right)}C_{k}\right)\Sigma_{k|k-1}^{\left(i\right)}. \end{array}$$

The likelihood of the motion model at step *k* is given by Equation (14). As the intention is determined for a period, let the number of steps that defines an intention be $N_{h}$ steps. The intention likelihood is defined and derived as the joint likelihood of the model likelihoods $L_{k}^{\left(i\right)}$:

(20) $$\begin{array}{cc} L_{k}\left(U^{\left(i\right)}\right)\; & =\prod_{\kappa =k-N_{h}+1}^{k}L_{k}^{\left(i\right)}\left(U^{\left(i\right)}\right)=\prod_{\kappa =k-N_{h}+1}^{k}{\left|2\pi S_{\kappa }^{\left(i\right)}\right|}^{-\frac{1}{2}}\exp\left[-\frac{1}{2}{\left({\tilde{z}}_{\kappa }^{\left(i\right)}\right)}^{⊤}{\left(S_{\kappa }^{\left(i\right)}\right)}^{-1}{\tilde{z}}_{\kappa }^{\left(i\right)}\right]. \end{array}$$

The control term that maximizes the intention likelihood is then selected:

(21) $$\begin{array}{c} i_{k}=\underset{i}{arg max}\left\{L_{k}\left(U^{\left(i\right)}\right)|\forall i\right\}, \end{array}$$

if the intention likelihood is above the threshold

$$\begin{array}{c} L_{k}\left(U^{\left(i_{k}\right)}\right)\geq L^{*}\left(U^{\left(i_{k}\right)}\right). \end{array}$$

The corresponding intention $\eta_{k}$ is given by

(22) $$\begin{array}{c} \eta_{k}=\left\{\begin{array}{cc} \eta^{\left(i_{k}\right)} & L_{k}\left(U^{\left(i_{k}\right)}\right)\geq L^{*}\left(U^{\left(i_{k}\right)}\right)\\ ø & \mathrm{Otherwise} \end{array}\right. \end{array}$$

where ø indicating an empty element means that there is no matching intention. The recursive operation infers intention for all steps, $\eta_{1:K}$.

#### 3.1.3. Intention-Pattern Modeling

The first process of the intention-pattern modeling, the extraction of the intended motions, checks the intention $\eta_{k}$ and identifies its period. Let the *j*th segment of the *i*th intention extracted from the smoothed state trajectory ${\overset{ˇ}{x}}_{1:K}$ be ${\overset{ˇ}{x}}_{k_{s}\left(i,j\right):k_{e}\left(i,j\right)}$. The second process of alignment aligns the extracted segments by co-locating their origins ${\overset{ˇ}{x}}_{k_{0}\left(i,j\right)}$:

$$\begin{array}{c} k_{0}\left(i,j\right)=0,\\ {\overset{ˇ}{x}}_{k_{0}\left(i,j\right)}={\overset{ˇ}{x}}_{k_{0}\left(i\right)}=\mathrm{const}., \end{array}$$

where the step of the origin $k_{0}\left(i,j\right)\in \left\{k_{s}\left(i,j\right),...,k_{e}\left(i,j\right)\right\}$.

**Figure 4:**
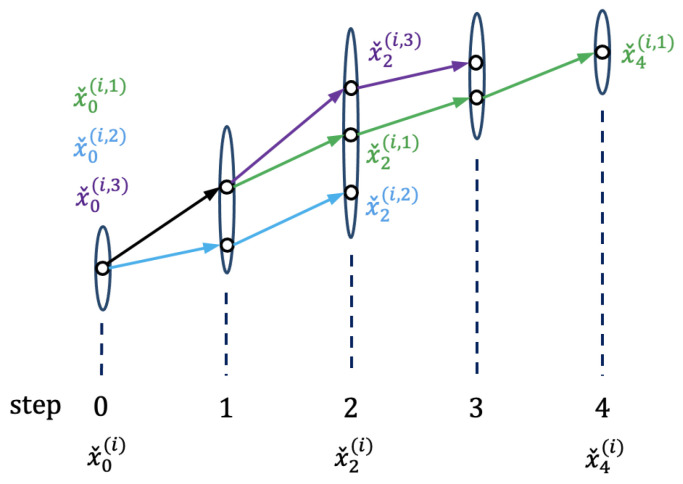
A set of Gaussian distributions representing the motion pattern which is the output of intention-pattern model.

The final process of motion pattern characterization derives the intention-pattern model by probabilistically characterizing the aligned segments. Figure 4 shows the characterization after three segments—green, blue, and purple—are aligned. As the number of segments increases, it is valid to assume that the variation of the motion follows a Gaussian distribution:

$$\begin{array}{c} {\overset{ˇ}{x}}_{\kappa }^{\left(i\right)}∼N\left({\bar{\overset{ˇ}{x}}}_{\kappa }^{\left(i\right)},{\overset{ˇ}{\Sigma }}_{\kappa }^{\left(i\right)}\right), \end{array}$$

where κ is a step of the intention-pattern model after alignment, and the mean and the covariance are

$$\begin{array}{c} {\bar{\overset{ˇ}{x}}}_{\kappa }^{\left(i\right)}=\frac{1}{n_{\kappa }^{\left(i\right)}}\sum_{j}{\overset{ˇ}{x}}_{\kappa }^{\left(i,j\right)},\\ {\overset{ˇ}{\Sigma }}_{\kappa }^{\left(i\right)}=\frac{1}{n_{\kappa }^{\left(i\right)}}\sum_{j}\left({\overset{ˇ}{x}}_{\kappa }^{\left(i,j\right)}-{\bar{\overset{ˇ}{x}}}_{\kappa }^{\left(i\right)}\right){\left({\overset{ˇ}{x}}_{\kappa }^{\left(i,j\right)}-{\bar{\overset{ˇ}{x}}}_{\kappa }^{\left(i\right)}\right)}^{⊤}. \end{array}$$

$n_{\kappa }^{\left(i\right)}$ is the number of segments at step κ for the *i*th intention [30]. The intention-pattern model is finally derived as

(23) $$\begin{array}{c} {\overset{ˇ}{x}}_{k}^{\left(i\right)}∼N\left({\bar{\overset{ˇ}{x}}}_{\kappa }^{\left(i\right)},{\overset{ˇ}{\Sigma }}_{\kappa }^{\left(i\right)}\right)\delta \left(k-\kappa \right), \end{array}$$

or

$$\begin{array}{c} {\bar{\overset{ˇ}{x}}}_{k}^{\left(i\right)}={\bar{\overset{ˇ}{x}}}_{\kappa }^{\left(i\right)}\delta \left(k-\kappa \right),\\ {\overset{ˇ}{\Sigma }}_{k}^{\left(i\right)}={\overset{ˇ}{\Sigma }}_{\kappa }^{\left(i\right)}\delta \left(k-\kappa \right), \end{array}$$

where δ(·) is a Dirac delta function. This means that the intention-pattern model is defined by a set of Gaussian distributions:

(24) $$\begin{array}{c} N^{\left(i\right)}=\left\{N\left({\bar{\overset{ˇ}{x}}}_{\kappa }^{\left(i\right)},{\overset{ˇ}{\Sigma }}_{\kappa }^{\left(i\right)}\right)|\forall \kappa \right\}. \end{array}$$

### 3.2. Estimation Using Intention-Pattern Model

**Figure 5:**
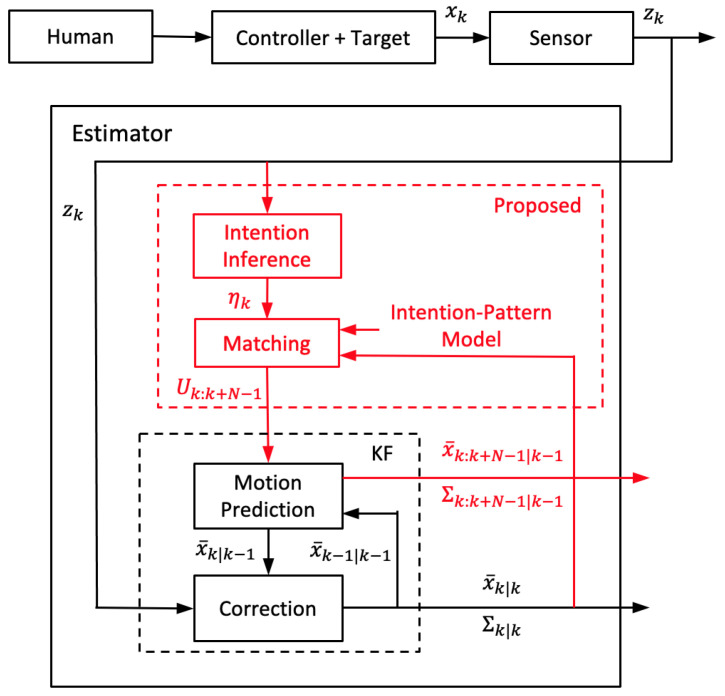
Estimation taking advantage of the proposed intention-pattern model. The red indicates the proposed parts compared with the conventional KF.

Figure 5 shows the schematics of the proposed state estimator using the intention-pattern model. Given a new observation $z_{k}$, Equations (19)–(22) output the intention of the current step, $\eta_{k}=\eta^{\left(i_{k}\right)}$. The estimator then checks the corresponding Gaussian distributions $N^{\left(i_{k}\right)}$ to find the matching step $\kappa_{k}$ with respect to the recent estimate state $x_{k-1|k-1}$:

(25) $$\begin{array}{c} L\left(x_{k-1|k-1}|N^{\left(i_{k}\right)}\right)=\max_{\kappa }Pr\left\{x_{k-1|k-1}|N\left({\bar{\overset{ˇ}{x}}}_{\kappa }^{\left(i_{k}\right)},{\overset{ˇ}{\Sigma }}_{\kappa }^{\left(i_{k}\right)}\right)\right\} \end{array}$$

(26) $$\begin{array}{c} \kappa_{k}=\underset{\kappa }{arg max}Pr\left\{x_{k-1|k-1}|N\left({\bar{\overset{ˇ}{x}}}_{\kappa }^{\left(i_{k}\right)},{\overset{ˇ}{\Sigma }}_{\kappa }^{\left(i_{k}\right)}\right)\right\}. \end{array}$$

The step $\kappa_{k}$ of $N^{\left(i_{k}\right)}$ is chosen if the intention-pattern model of the $i_{k}$th intention is satisfactory:

(27) $$\begin{array}{c} L\left(x_{k-1|k-1}|N^{\left(i_{k}\right)}\right)\geq L^{*}\left(N^{\left(i_{k}\right)}\right), \end{array}$$

and the step $\kappa_{k}$ matches the current step *k*.

Having the step $\kappa_{k}$ of the intention $i_{k}$ identified, the state is predicted by

(28a) $$\begin{array}{c} {\bar{x}}_{k|k-1}=A_{k}{\bar{x}}_{k-1|k-1}+{\bar{U}}_{k}^{\left(i_{k}\right)}, \end{array}$$

(28b) $$\begin{array}{c} \Sigma_{k|k-1}=A_{k}\Sigma_{k-1|k-1}{\left(A_{k}\right)}^{⊤}+T_{k}^{\left(i_{k}\right)}+Q_{k}. \end{array}$$

where the control term $U_{k}^{\left(i_{k}\right)}$ and the covariance of the control uncertainty, $T_{k}^{\left(i_{k}\right)}$, are derived from the intention-pattern model as

(29a) $$\begin{array}{c} {\bar{U}}_{k}^{\left(i_{k}\right)}={\bar{\overset{ˇ}{x}}}_{\kappa_{k}+1}^{\left(i_{k}\right)}-A_{k}{\bar{\overset{ˇ}{x}}}_{\kappa_{k}}^{\left(i_{k}\right)},\\ T_{k}^{\left(i_{k}\right)}=E\left[\left(U_{k}^{\left(i_{k}\right)}-{\bar{U}}_{k}^{\left(i_{k}\right)}\right){\left(U_{k}^{\left(i_{k}\right)}-{\bar{U}}_{k}^{\left(i_{k}\right)}\right)}^{⊤}\right]\\ =E\left\{\left[{\overset{ˇ}{x}}_{\kappa_{k}+1}^{\left(i_{k}\right)}-{\bar{\overset{ˇ}{x}}}_{\kappa_{k}+1}^{\left(i_{k}\right)}-A_{k}\left({\overset{ˇ}{x}}_{\kappa_{k}}^{\left(i_{k}\right)}-{\bar{\overset{ˇ}{x}}}_{\kappa_{k}}^{\left(i_{k}\right)}\right)\right]{\left[{\overset{ˇ}{x}}_{\kappa_{k}+1}^{\left(i_{k}\right)}-{\bar{\overset{ˇ}{x}}}_{\kappa_{k}+1}^{\left(i_{k}\right)}-A_{k}\left({\overset{ˇ}{x}}_{\kappa_{k}}^{\left(i_{k}\right)}-{\bar{\overset{ˇ}{x}}}_{\kappa_{k}}^{\left(i_{k}\right)}\right)\right]}^{⊤}\right\} \end{array}$$

(29b) $$\begin{array}{c} ={\overset{ˇ}{\Sigma }}_{\kappa_{k}+1}^{\left(i_{k}\right)}+A_{k}{\overset{ˇ}{\Sigma }}_{\kappa_{k}}^{\left(i_{k}\right)}A_{k}^{⊤}, \end{array}$$

Equation (28) shows that the covariance propagation is more than that of the conventional KF-based estimation by the addition of $T_{k}^{\left(i_{k}\right)}$. The correction is conducted by Equation (19c)–(19g) of KF with $i_{k}$ on behalf of *i*.

Because the proposed approach estimates the state incorporating the human intention, the mean of the estimated state is potentially more accurate than the conventional state estimation if observations are not available or reliable. The covariance of the estimated state is also more precise as it is updated adding the uncertainty of the human intention and prevents underestimation. Finally, note that the proposed state estimation allows future prediction with human intention in addition to the current estimation by recursively predicting the state with Equation (28).

## 4. Numerical Validation

Having the strength of the intention-pattern model identified, it is essential to test the proposed approach numerically and identify the capability and limitations. The approach was evaluated by applying to the state estimation of a human-maneuvered multirotor, which is one of the applications of this class with high demand. To identify the capability and limitations in depth, a simulated environment was created and used.

**Figure 6:**
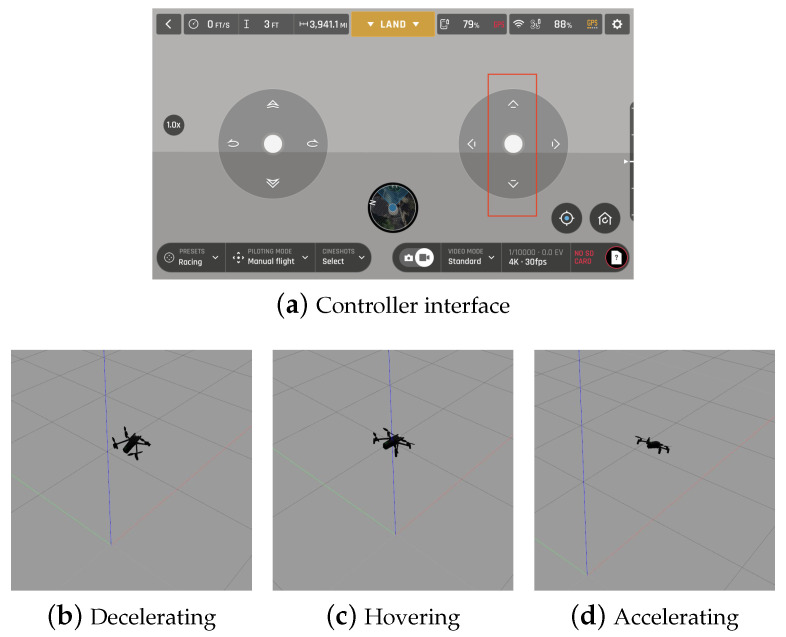
The controller interface of the SITL simulation environment and the motion examples of the multirotor for three intentions.

Figure 6 shows the controller interface used to create the multirotor motion and the resulting hovering, accelerating, and decelerating motions in the software-in-the-loop (SITL) simulation environment, whereas Table 1 lists the parameters used for simulation. With the right joystick of the controller interface, the human issues void command for hovering and forward or backward command for accelerating or decelerating.

**Figure 7:**
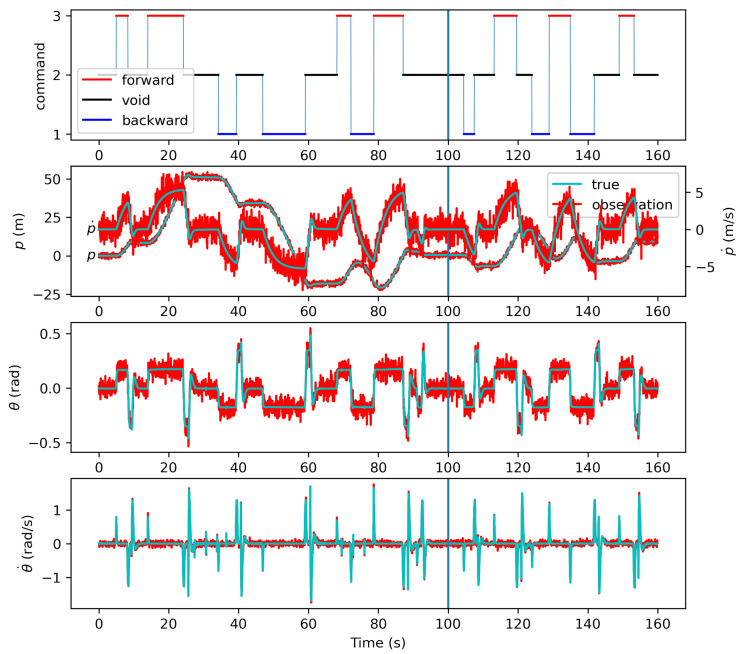
The human command and the true state and observation trajectories.

The multirotor dynamics was calculated in Gazebo, which also created motion noise artificially. As the most fundamental and typical motion, the linear horizontal motion of the multirotor was considered. The multirotor’s state, x, is given by

$$x={\left[p,\dot{p},\theta ,\dot{\theta }\right]}^{⊤}$$

where *p* is the position in the moving direction, $\dot{p}$ is the linear velocity, θ is the attitude (pitch angle), and $\dot{\theta }$ is the angular velocity. The estimator was assumed to observe all the state variables of the multirotor, i.e., $z={\left[z^{p},z^{\dot{p}},z^{\theta },z^{\dot{\theta }}\right]}^{⊤}$. The observations were created by adding noise to the true state where the noise variances are indicated as $\left[o_{s1}^{2},o_{s1}^{2},o_{s2}^{2},o_{s2}^{2}\right]$ as the variances are varied in the parametric study. Figure 7 shows the time-varying human command, true state and observation. The observation was created with $\left[o_{s1},o_{s2}\right]=\left[1,0.05\right]$. The observation noise was set high as the proposed approach is effective when the observation is uncertain or unavailable. The first 100 s was used to construct the intention-pattern model, and the state estimation using the proposed approach was conducted with the observation of the remaining 60 s. The command varies dynamically, and the multirotor motion is seen to reflect the commands of forward, void and backward.

Through the analysis of the multirotor state estimation problem, the motion and observation models used by the proposed approach were linear. The motion matrix $A_{k}$ is given by

(30) $$\begin{array}{c} A_{k}=\left[\begin{array}{cccc} 1 & dt & 0 & 1\\ 0 & 1 & 0 & 0\\ 0 & 0 & 1 & dt\\ 0 & 0 & 0 & 1 \end{array}\right]. \end{array}$$

whereas the observation matrix $C_{k}$ is a four-dimensional identity matrix. Table 2 lists the parameters of the proposed approach for both the intention-pattern model construction and the state estimation. The number of prediction steps between observations is denoted as $n_{p}$ as it takes a different value for each process/study. While the variances of the observation noise is known, those of the motion noise were determined from the theoretical and experimental analyses. $U^{\left(1\right)}$, $U^{\left(2\right)}$, and $U^{\left(3\right)}$ were chosen to infer the decelerating intention $\eta^{\left(1\right)}$, the hovering intention $\eta^{\left(2\right)}$, and the accelerating intention $\eta^{\left(3\right)}$, respectively. $\theta_{U}$ is a parameter to control the value of $U^{\left(i\right)}$ for parametric study.

Section 4.1 investigates the validity of the construction process of intention-pattern model through the parametric study. Section 4.2 then validates the estimation performance using the intention-pattern model.

### 4.1. Construction of Intention-Pattern Model

**Figure 8:**
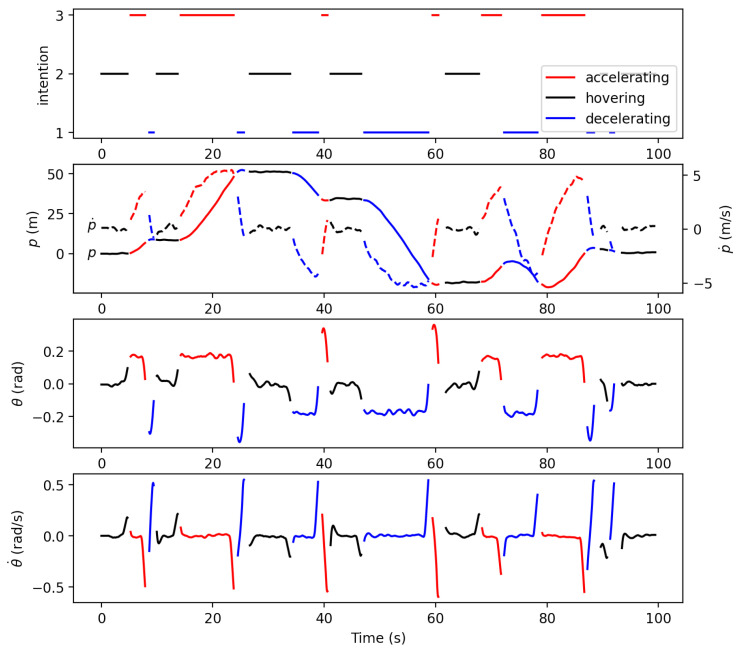
The inferred intention and the corresponding smoothed trajectory.

Figure 8 shows the inferred intentions and those in the corresponding smoothed trajectories when $\theta_{U}$ was 0.2. The smoothed trajectories are segmented based on the inferred intentions. The position is seen to appropriately increase and decrease when the human intention is with accelerating and decelerating respectively. As $U^{\left(1\right)}$, $U^{\left(2\right)}$, and $U^{\left(3\right)}$ differ from each other in the pitch angle θ, the pitch angle plot also shows intentions clearly: θ near 0 indicates hovering; positive θ with large magnitude indicates accelerating; negative θ with large magnitude indicates decelerating.

**Figure 9:**
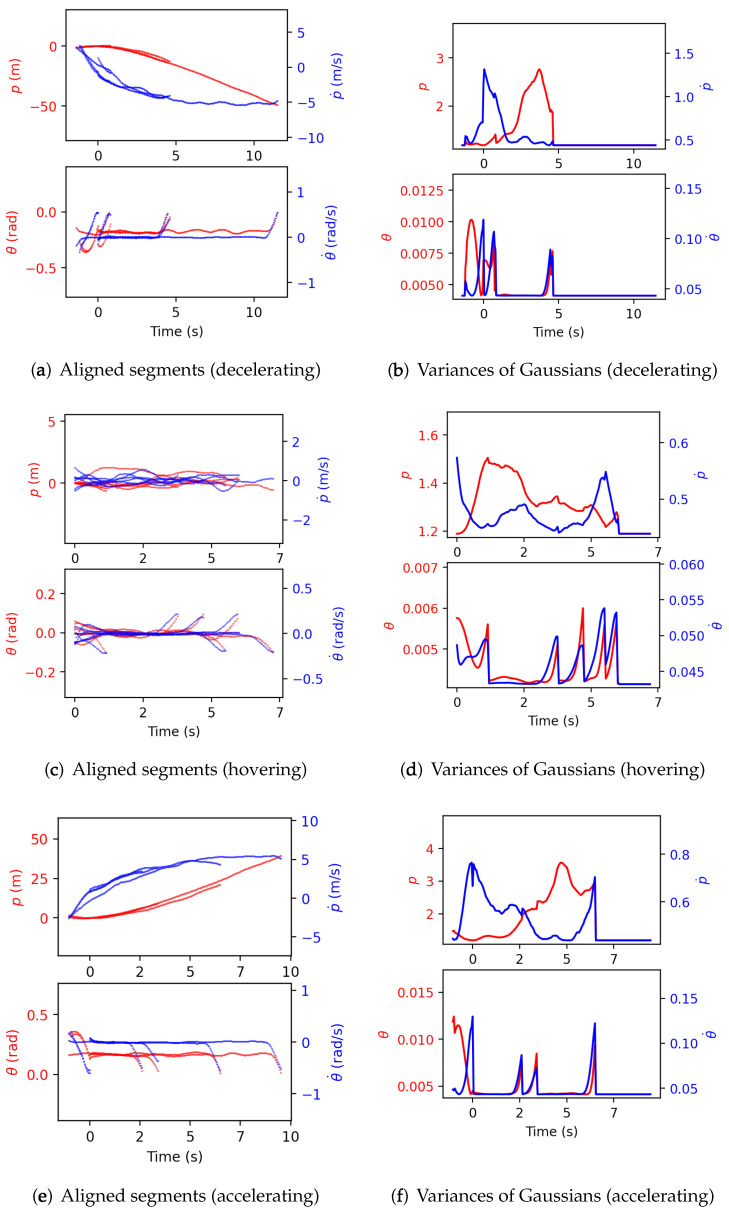
Construction of the intention-pattern model for three intentions.

Figure 9 shows the aligned segments and the variances of each resulting intention-pattern model when $n_{p}=1$. It is first seen that the aligned segments are consistent, which indicates that the proposed intention inference is valid. More consistency is shown in position than in pitch angle partly because the Gaussian assumption is not flexible enough to describe the pitch angle. The derived variances show that the intention-pattern models are modeled probabilistically from observations and could be used to perform state estimation more precisely.

**Figure 10:**
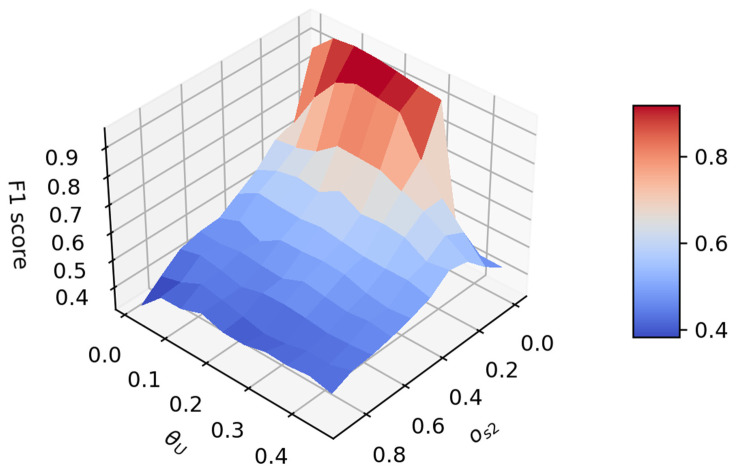
F1 score evaluating the intention inference accuracy with respect to simulation observation noise and the control term $U^{\left(i\right)}$.

To analyze the dependency of the intention inference, the F1 score [31], evaluating the inference performance was derived with different levels of observation noises and control terms. The parameters varied were $o_{s2}$ for the observation noise and $\theta_{U}$ for the control term as the pitch angle θ characterizes the intention. The ground truth intention was defined based on the real θ value: hovering when |θ|≤0.05; accelerating when θ>0.05; decelerating when θ<−0.05. The F1 score is calculated as

$$\begin{array}{c} \frac{2}{\frac{\mathrm{TP}+\mathrm{FP}}{\mathrm{TP}}+\frac{\mathrm{TP}+\mathrm{FN}}{\mathrm{TP}}} \end{array}$$

where TP, FP, and FN correspond to the number of steps of true positive, false positive, and false negative [31]. The F1 score which is closer to 1 indicates better inference. Figure 10 shows the distribution of the F1 score over $o_{s2}$ and $\theta_{U}$. As seen from the figure, the smaller the noise, the better the inference. For $\theta_{U}$, there is a best value in the middle; either too large or too small will result in poor inference.

**Figure 11:**
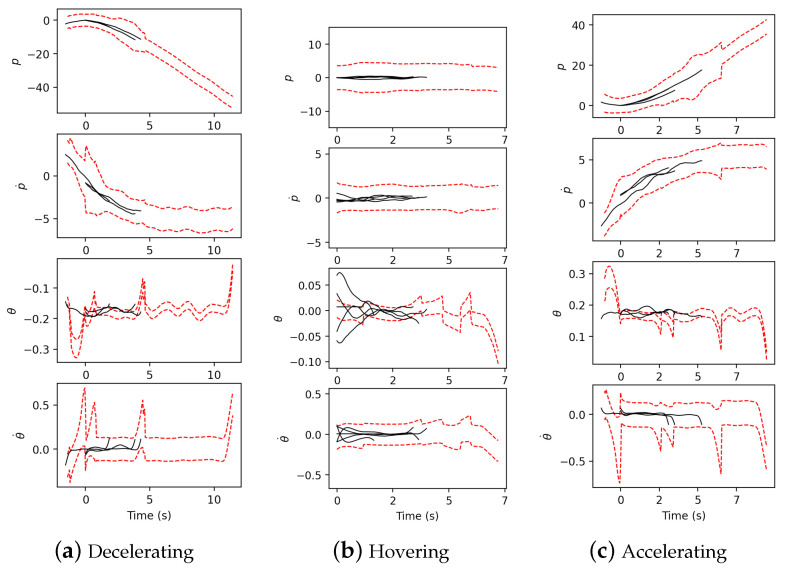
Validation of the constructed intention-pattern model.

Figure 11 shows the resulting performance of each intention-pattern model. The two red broken lines show the range of motion pattern defined by the variance of the intention-pattern model constructed from the first 100 s whereas the solid black lines the motions of the same intention-pattern model identified in the next 60 s. It is seen that motions extracted in the 60 s are well along with the range of the intention-pattern model. This verifies the validity of the probabilistically represented intention-pattern model.

### 4.2. Estimation Using Intention-Pattern Model

**Figure 12:**
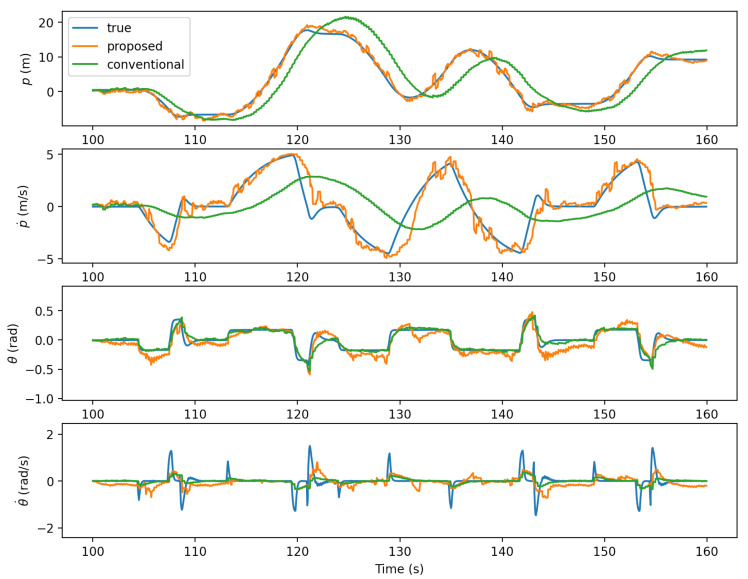
State estimation by the proposed and the conventional approaches.

Having the intention-pattern model constructed using the first 100 s, Figure 12 shows the result of state estimation incorporating the constructed intention-pattern model in the subsequent 60 s. Unlike the intention-pattern model, the state estimation uses $n_{p}=5$ as the effect of the proposed approach can be seen with the motion prediction. The ground truth and the result of the conventional KF estimation without intention incorporation are also shown for comparison. The estimation result of the proposed approach is seen to be closer to the ground truth than that of the conventional approach. The estimation of *p* and $\dot{p}$ particularly shows the responsive estimation of the proposed approach when the target motion is changed by the human while the conventional estimation exhibits notable delay. The faster response is due to the use of the intention-pattern model. The conventional approach could improve estimation by frequent accurate observation, but observations are often uncertain or unavailable.

**Figure 13:**
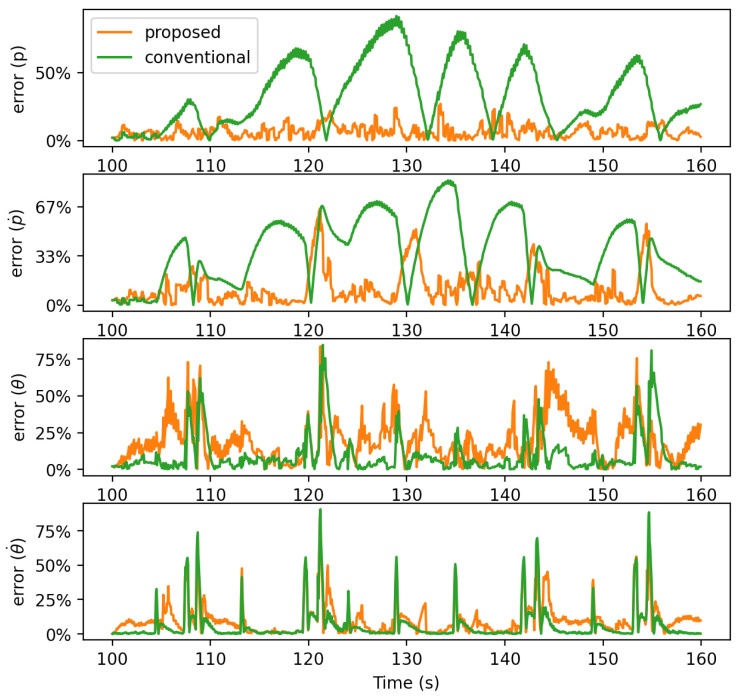
Absolute error of mean estimated by the proposed and the conventional approaches.

Figure 13 shows the absolute error of estimated mean of each state variable with respect to time. While seeing less difference in θ and $\dot{\theta }$, the error of the proposed approach in *p* and $\dot{p}$ consistently and significantly stays low compared to the conventional approach. The difference is particularly large when the human changes the target motion as the conventional approach does not take the human intention into account. The maximum error and the mean squared error (MSE), integrating the absolute errors to a single quantity, are improved by almost three times and 8.7 times, respectively, when the proposed approach was deployed.

**Figure 14:**
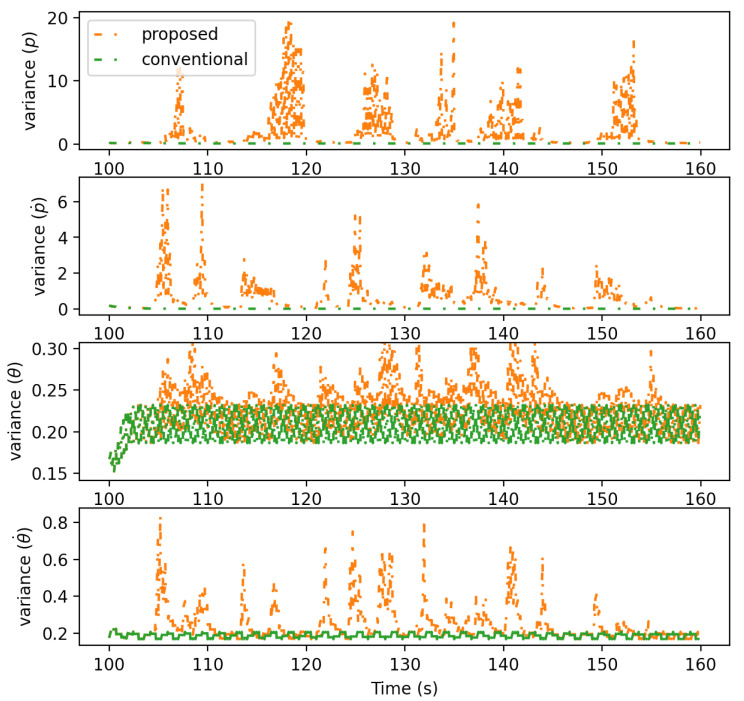
Variance estimated by the proposed and the conventional approaches.

Figure 14 shows the variance of each state variable estimated by the proposed and the conventional approaches. The result shows that the proposed approach exhibits larger variances than those of the conventional approach when the error is large. As the proposed approach infers human intentions and adds their uncertainties, its variance is estimated more precisely and adequately. The variance of the conventional approach, on the other hand, is significantly smaller though the mean estimation is wrong. Having the human control deterministically treated without inferring intentions, the uncertainty of the conventional approach is markedly underestimated.

**Figure 15:**
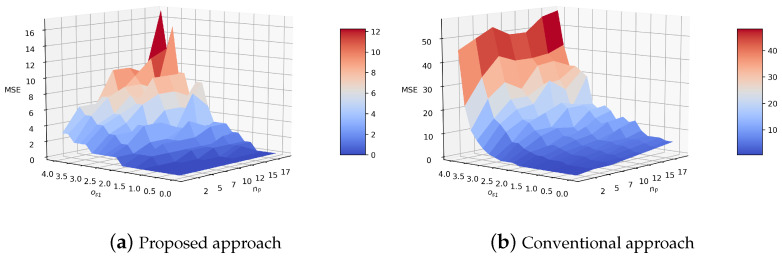
MSE with different observation noise and the number of prediction steps between observations.

The performance of the proposed approach in state estimation was further examined through the parametric study. Figure 15 shows the MSE of the proposed approach when $o_{s1}$ and $n_{p}$ were varied. $o_{s1}$ was varied to examine the effect of the observation noise as it contributed less at the construction of the intention-pattern model. The result of the conventional approach is also shown for comparison. It is first seen that the MSE of the proposed approach is significantly lower than that of the conventional approach when $o_{s1}$ is large. The large $o_{s1}$ increases the dependency of state estimation onto the prediction. As a result, the proposed approach, incorporating human intention and effective in prediction, can thus keep the MSE low. The result also shows that the MSE of the proposed approach remains low even when $n_{p}$ is large. $n_{p}$ also increases the dependency of state estimation onto the prediction, so the proposed approach becomes better than the conventional approach in accuracy. Meanwhile, the proposed and the conventional approaches exhibit a similar MSE when $o_{s1}$ is low and $n_{p}$ is one. This is because the estimation becomes correction-driven as the frequency of the accurate correction becomes high.

## 5. Conclusions

This paper has presented an approach that estimates the state of a human-maneuvered target incorporating human intention, which consists of a preprocess constructing an intention-pattern model, and the main process allowing state estimation using the intention-pattern model. The preprocess constructs the intention-pattern model from the prior observations and probabilistically represents the model. The main process, then, uses standard state estimation such as KF extensively leveraging the probabilistically represented intention-pattern model. In the application of the proposed approach to the state estimation of a human-maneuvered multirotor, the numerical result has first shown that the constructed intention-pattern model represents the human intention appropriately. The result of state estimation of the human-maneuvered multirotor then shows that the proposed approach estimates the state more accurately than the conventional approach particularly when observations are uncertain or unavailable. The proposed approach has also demonstrated that it can estimate the covariance more precisely.

The paper has reported the first progress of the state estimation of a human-maneuvered target using human intention, and much future work is possible. Ongoing work includes the extension of the proposed approach for partially observable problems and model predictive control. Observations are necessary for the construction of the intention-pattern model, but the state may not be fully observable. The proposed approach is effective in prediction-driven estimation, so the model predictive control of an autonomous robot becomes one of the most effective extensions. The outcomes will be summarized and published in the form of papers as soon as they are ready.

## Figures and Tables

**Table 1:** Parameters for simulation.

Parameter	Value
Human commands	Void, Forward, Backward
Simulation motion noise	Specified in Gazebo
Simulation observation noise variances	$\left[o_{s1}^{2},o_{s1}^{2},o_{s2}^{2},o_{s2}^{2}\right]$
Cruising inclination limit [rad]	0.17

**Table 2:** Parameters of the proposed approach.

Parameter	Value
Time step [s]	Δt=0.05
Number of predictions between two observations	$n_{p}$
Variances of motion noise Q	$\left[0,{\left(1-o_{s1}/4\right)}^{2}\Delta t^{2},0,2^{2}\Delta t^{2}\right]$
Variances of observation noise R	$\left[o_{s1}^{2},o_{s1}^{2},o_{s2}^{2},o_{s2}^{2}\right]$
Human intention	Decelerating, Hovering, Accelerating
$U^{\left(1\right)}$	${\left[0,0,-{\bar{x}}_{k-1|k-1}^{\left(i\right)}\left[3\right]-\theta_{U},0\right]}^{⊤}$
$U^{\left(2\right)}$	${\left[0,0,-{\bar{x}}_{k-1|k-1}^{\left(i\right)}\left[3\right],0\right]}^{⊤}$,
$U^{\left(3\right)}$	${\left[0,0,-{\bar{x}}_{k-1|k-1}^{\left(i\right)}\left[3\right]+\theta_{U},0\right]}^{⊤}$
Smoothing technique	Triangular moving average
Duration of construction [s]	100
Duration of estimation [s]	60
